# Reproductive injury in male BALB/c mice infected with *Neospora caninum*

**DOI:** 10.1186/s13071-021-04664-y

**Published:** 2021-03-16

**Authors:** Hang Li, Bing-Yi Yang, Ming-Ming Liu, Shao-Wei Zhao, Su-Zhu Xie, Hao Wang, Shuang Zhang, Xue-Nan Xuan, Li-Jun Jia

**Affiliations:** 1 Engineering Research Center of North-East Cold Region Beef Cattle Science & Technology Innovation, Ministry of Education, Yanbian University, No.977 Park Road, Yanji, 133002 People’s Republic of China; 2 National Research Center for Protozoan Diseases, Obihiro University of Agriculture Veterinary Medicine, Inada-cho, Obihiro, Hokkaido 080-8555 Japan

**Keywords:** *Neospora caninum*, Male, BALB/c mice, Reproductive system, Injury

## Abstract

**Background:**

*Neospora caninum* is one of the main causes of abortion in pregnant animals. However, *N. caninum*-induced reproductive injury in male mice is still unclear.

**Methods:**

Male BALB/c mice were infected with a bovine isolate of *N. caninum*, and the organ coefficients of the testis and epididymis were measured. Lesions in the testis and epididymis were observed by light microscopy and transmission electron microscopy. Expression of the spermatogenic cell apoptosis-related proteins p53 and caspase-3 was detected by western blot. The expression of spermatogenesis-related genes in the testis was detected by reverse transcription-PCR. Sperm morphology and motility were observed. The levels of nitric oxide (NO) and antisperm antibody (AsAb) in the testicular homogenates and hormones in the serum were detected by enzyme-linked immunosorbent assay. The reproductive capacity of the male mice was detected using a reproduction test.

**Results:**

The organ coefficients of the testis and epididymis of the experimental group were significantly downregulated. Light microscopy examination revealed that the spermatogenic cells of the testis were arranged in a disordered manner, and the number was reduced. The number of sperm in the epididymal lumen was significantly reduced, and the cytoplasm exhibited vacuolation and degeneration. Ultrastructural studies revealed that the cells of the testis and epididymis tissues showed varying degrees of disease. The level of p53 and caspase-3 expression in the testis was significantly upregulated. The expression of the testicular spermatogenesis-related genes Herc4, Ipo11 and Mrto4 were strongly downregulated. Observation of sperm by microscopic examination revealed significantly reduced sperm density and sperm motility, and the number of sperm deformities was significantly increased. The level of NO and AsAb was significantly increased. The levels of luteinizing hormone, follicle-stimulating hormone and gonadotropin-releasing hormone were significantly upregulated, whereas the levels of testosterone, thyrotropin-releasing hormone, thyroxine and thyroid-stimulating hormone were significantly downregulated. After challenge, the infected male mice and healthy female mice were caged together: the subsequent fetal death rate was increased, and the conception rate, litter size, number of live births and the birth weight were significantly reduced.

**Conclusions:**

Infection of male BALB/c mice with the bovine isolate of *N. caninum* induced varying degrees of injury to the testis, epididymis and sperm of the mice, destroyed spermatogenesis and affected the reproductive capacity.
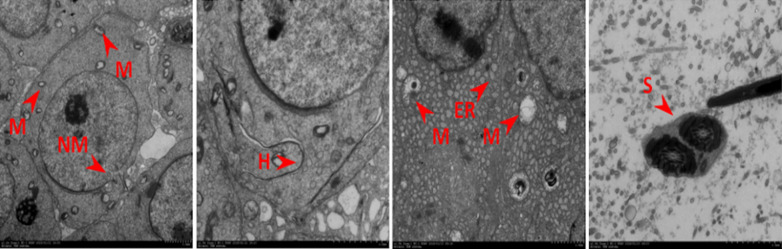

## Background

*Neospora caninum* is morphologically classified as a protozoa. It has three predominant life-cycle stages, namely tachyzoite, cyst and oocyst, and it is spread in livestock herds through oocysts [1]. While dogs are the final host of *N. caninum*, intermediate hosts include cattle, horses, sheep, pigs, deer, chickens, pigeons and other animals. Once an animal is infected, * N. caninum* is able to parasitize the brain, liver, spleen, placenta and other tissues and organs, causing tissue damage.

*Neospora caninum* can be transmitted to new hosts through horizontal and vertical transmission [2]. Some researchers have found that *N. caninum* can be vertically transmitted to the fetus in infected pregnant rhesus macaques [3]; however, there is as yet no direct evidence that *N. caninum* can infect humans [4]. Based on the immune response to *N. caninum* infection in pregnant ewes, controlling placental transmission and fetal abortion is related to the intensity of the cell-mediated immune response during pregnancy [5]. In 2003 and 2004, researchers detected *N. caninum* DNA in the semen of naturally infected bulls, which indicated the possibility of sexual transmission of *N. caninum* [6]. Bahrami et al. diagnosed 30 cattle infected with *N. caninum* and 15 healthy cattle by the modified Rose bengal plate test and enzyme-linked immunosorbent assay (ELISA) and studied sperm parameters [7]. The results of this study showed that the concentration, viability and motility of sperm were significantly lower in bulls with neosporosis [7]. To date, there have been few reports of the effects of *N. caninum* on the reproductive organs of male mice.

Recent research on *N. caninum* has made some progress, but the pathogenic mechanism remains unclear, especially in terms of *N. caninum*-induced injury to the reproductive system of male animals. Little information is available on whether *N. caninum* can be transmitted from males to females and subsequently to their offspring. Therefore, in view of the major harm caused by *N. caninum* to livestock reproduction and challenges that need to be urgently resolved, the aim of this study was to establish a male BALB/c mouse model of infection with a bovine isolate of *N. caninum*. We employed hematoxylin and eosin (HE) staining, transmission electron microscopy (TEM), Western blot, reverse transcription (RT)-PCR and ELISA to elucidate the damaging effect of *N. caninum* on the reproductive system of male mice. The results provide a basis for the study of the pathogenic mechanism of *N. caninum*.

## Methods

### Experimental animals

Eight-week-old female and male BALB/c mice, each mouse weighing approximately 20 g, were purchased from the Liaoning Changsheng Experimental Animal Co. Ltd., China (Certificate No. SCXK [Liao] 2015-0001). The mice were domesticated under controlled temperature (25 ± 1 ℃) and humidity (50 ± 5%) and had free access to food and water. All animal experiments started 1 week after habituation. The recommendations in the Guide for the Care and Use of Laboratory Animals of Yanbian University, Yanji, China, were strictly followed. The protocol of the present study was approved by the Committee on the Ethics of Animal Experiments of Yanbian University (permission 20180301).

### Culture and purification of the bovine isolated *N. caninum*

The bovine isolate of *N. caninum* was obtained from the brain tissue of bovine fetuses and cultured at the Yanbian University Preventive Veterinary Laboratory, Yanji, China [8]. Cells of the African green monkey kidney cell line (Vero) were preserved at the Preventive Veterinary Laboratory of Yanbian University.

*Neospora caninum* parasites were propagated in Vero cells and cultured in Dulbecco’s Modified Eagle’s medium (DMEM; Sigma-Aldrich, St. Louis, MO, USA). Subsequently, the cells were supplemented with 8% heat-inactivated fetal bovine serum (Gibco Laboratories, Gaithersburg, MD, USA), 100 g/ml penicillin and 10 mg/ml streptomycin (Solarbio, Beijing, China), at 37 ℃ in a 5% CO_2_ atmosphere. Tachyzoites were purified from the infected Vero cells by washing the cells in ice-cold phosphate-buffered saline (PBS; Solarbio), followed by three passages through a 27-gauge needle syringe. The tachyzoites were subjected to filtration through a 5.0-μm pore filter to remove the host cell debris and centrifuged at 600  *g* for 10 min. Parasite numbers were counted with a hemocytometer.

### Grouping of experimental animals and establishing animal models

A total of 80 BALB/c mice were randomly selected, of which 60 were males and 20 were females. To detect various indicators after establishing animal models (*n* = 40), 20 male mice were randomized to the experimental group and 20 male mice were randomized to the control group. For the reproduction test (*n* = 40), the experimental group consisted of ten challenged male mice and ten healthy female mice (*n* = 10); the control group consisted of ten healthy male mice and ten healthy female mice.

A male BALB/c mouse model of *N. caninum* was established according to the methods described by Muller [9] and Wang [10]. Briefly, each mouse in the experimental group was intraperitoneally inoculated with 10^5^ tachyzoites diluted in 200 µl of ice-cold PBS. The clinical symptoms of the mice were observed daily.

### Detection of the reproductive organ coefficient

At 14 days post-infection, the blood was collected by extirpating the eyeball, and the mice were euthanized by cervical vertebra luxation. Blood samples were obtained and centrifuged, and the serum was retained to test hormone levels. Testis and epididymis tissues were collected aseptically; following removal of the fat and connective tissue, these tissues weighed and the reproductive organ coefficient was determined according to the organ index measurement method: reproductive organ coefficient (left testis or epididymis) = weight of reproductive organ (left testis or epididymis) per mouse/body weight per mouse (mg/g) [11]. After measuring the organ coefficient, other testis and epididymis experiments were performed. For each experiment, five experimental group samples and five control group samples were used.

### Histopathological observations of the changes in reproductive organs

The testis and epididymis tissues were put into the 10% neutral formalin for 12 h, following which they were dehydrated using a standard procedure, embedded in paraffin, sliced into 5-μm sections, adhered onto a slide covered with poly-lysine and placed into an oven at 80 ℃ for 1 h. The slices were then dewaxed in xylene, rehydrated through decreasing concentrations of ethanol and washed in PBS before staining with HE. After staining, the slices were dehydrated through increasing concentrations of ethanol and xylene, then covered by general clarity gum. The samples were observed by light microscopy (model BX43 microscope; Olympus Corp., Tokyo, Japan).

### TEM observations

The testis and epididymis tissues were fixed in 4% glutaraldehyde for 1 week at 4 ℃, followed by treatment in 1% osmic acid for 2 h at 4 ℃. The tissue blocks were then dehydrated in a series of graded ethanol (50, 70, 80 and 95%, 10 min at each concentration), followed by treatment in anhydrous alcohol: anhydrous acetone (1:1) for 10 min and in anhydrous acetone for 20 min. Once the tissue were embedded in araldite, ultrathin slices were stained with uranium dioxide acetate and lead citrate for 10 min [12]. The tissues were observed by TEM (model H-7650 microscope; Hitachi Ltd., Tokyo, Japan).

### Western blot detection of p53 and Caspase-3 expression in the testis

A volume of 1 ml RIPA lysate was added to every 0.1 g of tissue (Biyuntian Company, Shanghai, China). Protein concentrations were measured using a BCA Protein Assay Kit (Thermo Scientific, Waltham, MA, USA). A 30-μg sample of each protein was separated by 12% sodium dodecyl sulfate–polyacrylamide gel electrophoresis (SDS-PAGE) and then transferred to polyvinylidene fluoride membranes (Millipore, Bedford, MA, USA) by the wet transfer method. After 2 h of blocking in TBS-0.05% Tween 20 containing 5% skim milk, the membranes were incubated at 4 ℃ overnight with monoclonal mouse antibodies, p53, caspase-3 (1:1000 dilution, respectively) and β-actin (1:3000 dilution) (Zhongshan Golden Bridge, Beijing, China). Horseradish peroxidase (HRP)-conjugated goat anti-rabbit antibodies (1:2000 dilution) (Zhongshan Golden Bridge) were used as secondary antibodies at room temperature for 1 h. Finally, the membranes were washed three times with PBS-Tween, and the signals were detected with enhanced chemiluminescence. Densitometry analyses of all bands were performed using Image Lab software (Bio-Rad, Richmond, CA, USA).

### Detecting the expression of testicular spermatogenic function-related genes* Herc4*,* Ipo11* and* Mrto4* by RT-PCR

RNA was extracted using TRIzol reagent (Invitrogen, Carlsbad, CA, USA). All RNA precipitates were dissolved in 20 µl of nuclease-free water, and the RNA concentration and integrity were determined using a NanoDrop 2000 instrument (Thermo Scientific, Waltham, MA, USA). The cDNA synthesis was performed using 2 µg of total RNA in a final volume of 20 µl using a PrimeScript™ RT Reagent Kit (TaKaRa, Dalian, China) according to the manufacturer’s instructions. RT-PCR was performed using the qTOWER 2.2 system (Biometra, Analytik Jena AG, Jena, Germany) with the following steps: 95 ℃, 15 min; then 95 ℃/10 s and 60 ℃/32 s for 40 cycles. The data were normalized to the expression of β-actin. All primers were synthesized by Sangon Biotech (Shanghai, China), and the nucleotide sequences of the primers are presented in Table 1. The data were analyzed based on raw cycle thresholds (Cq), obtained from the ABI Prism 7500 software using the comparative Cq method (2^−ΔΔCq^ method). The relative level of *Herc4*, *Ipo11* and *Mrto4* expression was calculated using the 2^−ΔΔCt^ formula, where ΔΔCt represented the Ct (sample) − Ct (control). Every sample was tested in triplicate, and the data from three independent experiments were used for the final analysis.

**Table 1:** The primer sequences used for reverse transcription-PCR

mRNA species	Primer sequence
β-actin	Forward 5′-TCCAGCCTTCCTTCTTGGGT-3'
	Reverse 5′-GCACTGTGTTGGCATAGAGGT-3'
Herc4	Forward 5′-GCCTATGGAGTGTTGGCAGA-3'
	Reverse 5′-GACTGCATCTGTCTGGAGCA-3'
Mrto4	Forward 5′-ACCTGATAGAAGAGCTTCGGA-3′
	Reverse 5′-TCCTTC GTGCGGTTGGTAAA-3'
Ipo11	Forward 5′-TGTCTCAACACGAGGAACCG-3'
	Reverse 5′-CCTTCAGGGCCAGCATCTTT-3'

### Sperm morphological observations and motility statistics

After the mice had been euthanized, the epididymides were immediately removed and placed in a warm culture dish containing 2 ml Hank's balanced salt solution free of calcium and magnesium, supplemented with 0.2% bovine serum albumin (Sigma-Aldrich). The tissue was minced and placed in an incubator at 34 ℃ for 15 min. At least ten fields were observed under a light microscope at 400× magnification, and the percentage of sperm motility was recorded. After removing the cover glass, the sperm suspension was air-dried and fixed in methanol. A total of 300 sperm in different fields of each sample were observed and the deformity rate was counted. The sperm density was calculated using a hemocytometer.

### Detection of nitric oxide and antisperm antibody levels

The testicular tissue was weighed according to the proportion of mass:volume = 1:9, and normal saline was added and the tissue macerated. The tissue was then centrifuged at 1200  *g*, 4 ℃, for 10 min, and the supernatant diluted to 10% testis homogenate. The level of nitric oxide (NO) and antisperm antibody (AsAb) was measured using a mouse NO and AsAb ELISA Ready-SET-Go Kit (Alpha Diagnostic Intl Inc., San Antoinio, TX, USA). Briefly, 50 µl of standards and samples were carefully added to the sample wells, after which 100 µl of horseradish peroxidase (HRP)-labeled detection antibody was added; the contents of the wells were thoroughly mixed by swirling gently and incubated at 37 °C for 60 min. After a sufficiently long incubation, 100 µl of chromogenic substrate was added to each reaction and incubated for 15 min at 37 ℃ in the dark. The reactions were terminated by adding 50 µl of sulfuric acid and swirling gently to mix the wells. The ELISAs were read at 450 nm, and the obtained optical density (OD) values were converted by extrapolation using a standard curve.

### Detection of hormone levels

Serum collected from mice were analyzed with the ELISA Ready-SET-Go kit (Alpha Diagnostic Intl Inc.) for mouse gonadotropin-releasing hormone (GnRH), follicle-stimulating hormone (FSH), lutenizing hormone (LH), testosterone (T), thyrotropin-releasing hormone (TRH), thyroid-stimulating hormone (TSH) and thyroxine (T4). The specific detection steps were the same as those in the NO and AsAb detection methods, and the enzyme-labeled antibodies were anti-GnRH, anti-FSH, anti-LH, anti-T, anti-TRH, anti-TSH and anti-T4 antibodies. The assays were read at 450 nm, and the OD values obtained were converted by extrapolation using a standard curve.

### Reproduction test

Ten male mice that had been challenged for 14 days in the experimental group and ten male mice in the control group were caged with healthy female mice at a ratio of 1:1; females were estrus-synchronized by the Whitten effect. After 3 days, males were removed from the cages. The mice gave birth on days 20–22 post-mating. We counted the conception rate and fetus death rate, the number of F1 offspring, sex ratio, average body weight and other indicators to determine the effect of *N. caninum* on the reproductive ability of male mice.

### Statistical analysis

All values are expressed as the mean ± standard deviation (SD). A one-way analysis of variance (ANOVA) was used. All statistical analyses were performed using SPSS version 20.0 statistical analysis software (IBM Corp., Armonk, NY, USA). Differences between test groups were considered significant if the *P* value was ≤  0.05.

## Results

### Establishment of a male BALB/c mouse model of *N. caninum*

**Fig. 1:**
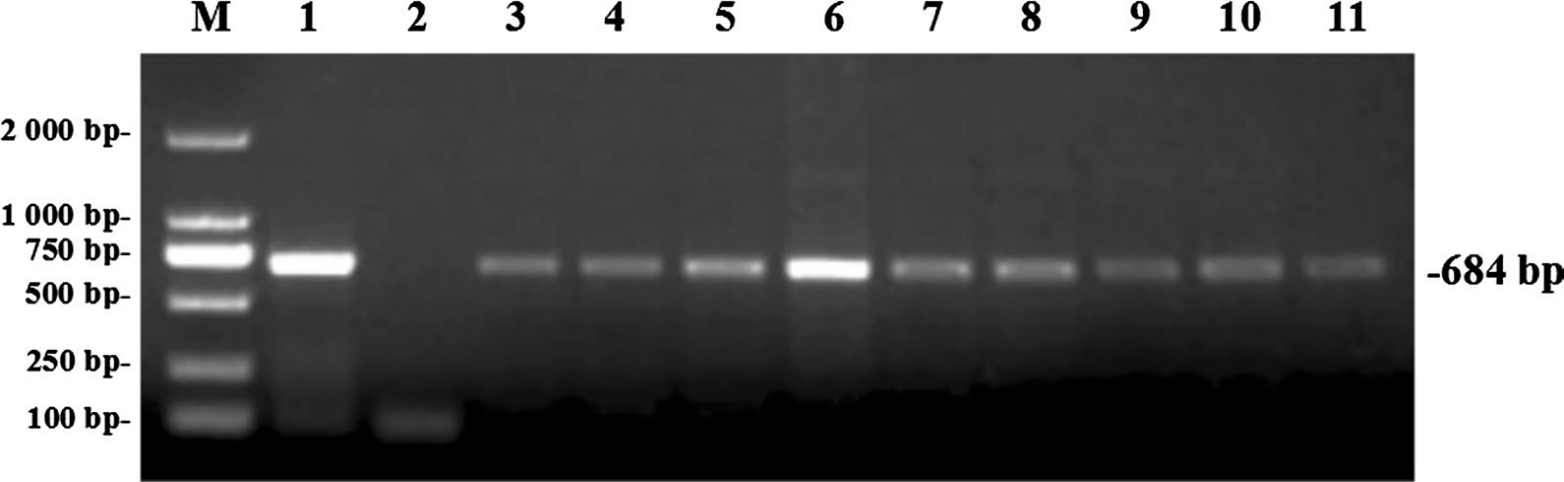
PCR detection of the* Nc-5* gene of *Neospora caninum* in organs of challenged male BALB/c mice. Lanes: *M* DL2000 DNA marker,* 1* positive control,* 2* negative control,* 3* heart,* 4* liver,* 5* spleen,* 6* brain,* 7* lung,* 8* kidney,* 9* testis,* 10* epididymis,* 11* semen

Challenged male BALB/c mice showed typical clinical symptoms of *N. caninum* infection (e.g. loose hair and ataxia). After 14 days, the* Nc-5* gene of *N. caninum* was detected in the heart, liver, spleen, lung, kidney, brain, testis, epididymis and semen by PCR (Fig. 1). This finding indicated that the established male BALB/c mouse model of *N. caninum* met the test requirements.

### Effect of *N. caninum* on the reproductive organ coefficient

**Fig. 2:**
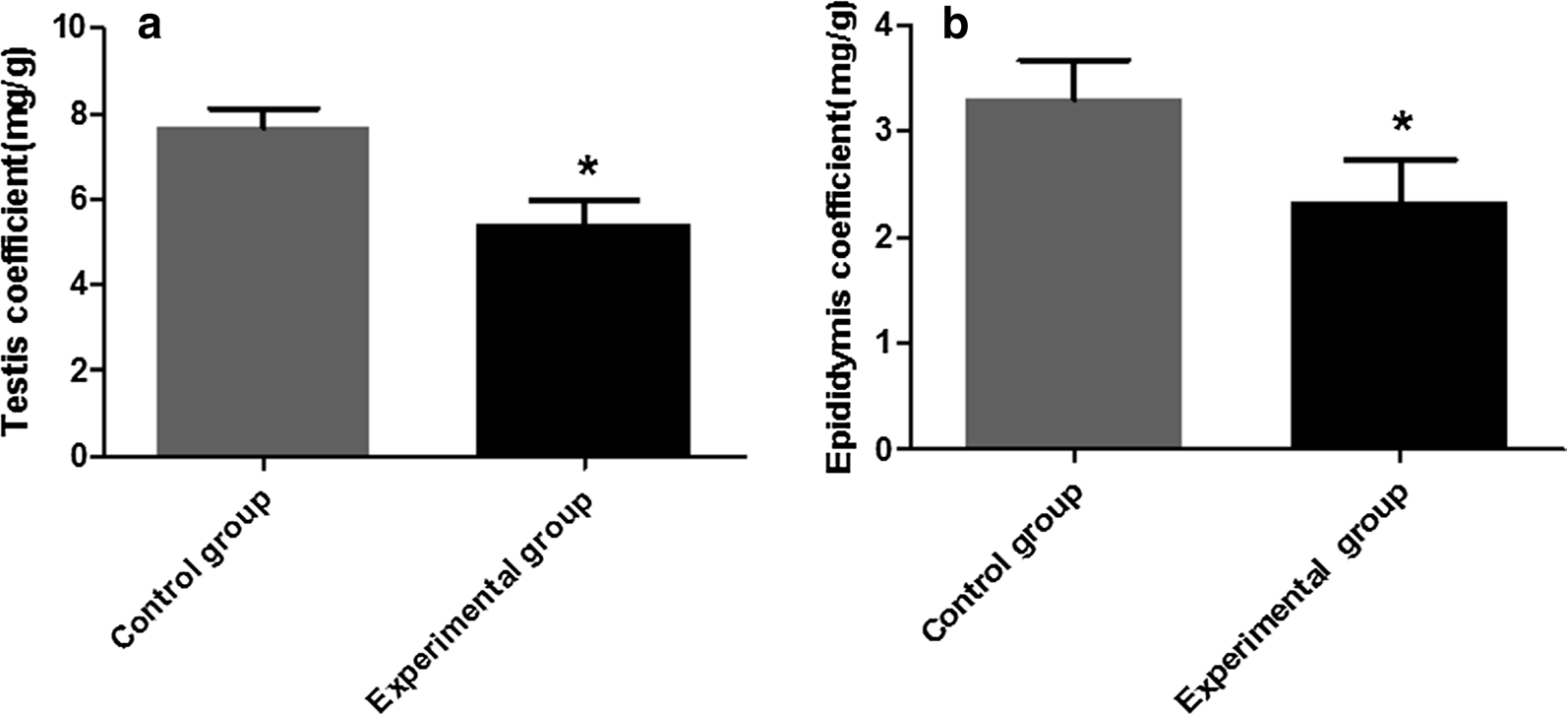
Coefficients of the reproductive organs of challenged male BALB/c mice. **a** Testis coefficient, **b** epididymis coefficient. The data are presented as the mean ± standard error of the mean (SEM). Asterisk indicates that the difference between coefficients for the experimental group and control group is statistically significant at *P* < 0.05

The reproductive organ coefficient (testis or epididymis) was determined as defined in the Methods section. After statistical analysis, the organ coefficients of testis (ANOVA: *F*_(1, 8)_ = 10.87, *P* = 0.011) and epididymis (ANOVA: *F*_(1, 8)_ = 10.72, *P* = 0.011) were recorded to be downregulated compared with the control group (Fig. 2a, b).

### Histopathological observations of testicular and epididymal lesions

**Fig. 3:**
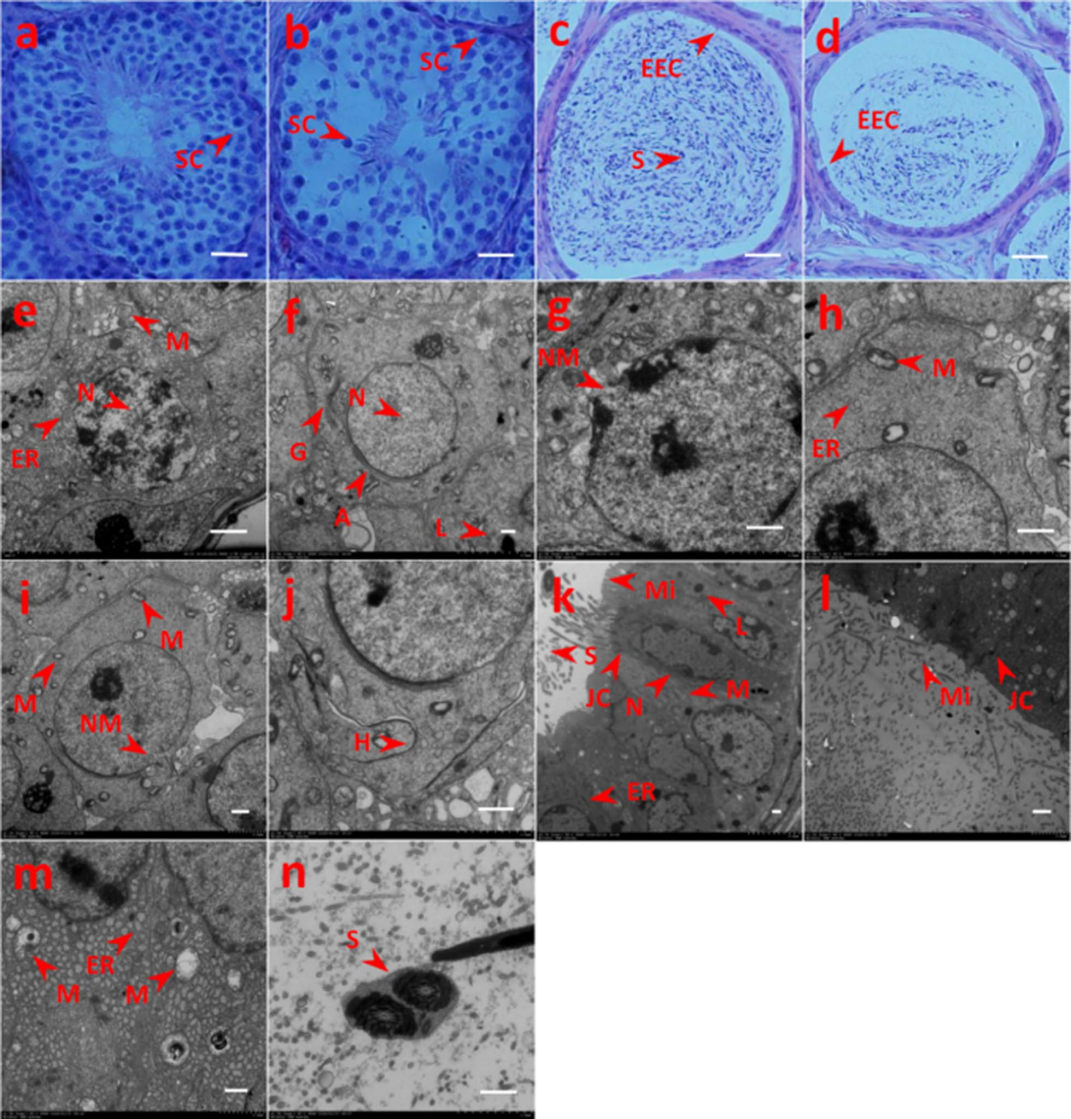
Light microscropy (with hematoxylin and eosin [HE] staining) and transmission electron microscopy (TEM) observation of testis and epididymis of challenged and control male BALB/c mice. **a**, **b** HE staining of testis in control (non-infected) (**a**) and challenged (**b**) male mice: **a** the number and stucture of spermatogenic cells in control mice were normal, **b** the number of spermatogenic cells in challenged mice was significantly reduced and seriously exfoliated. **c**, **d** HE staining of epididymis in control (**c**) and challenged (**d**) mice: **c **the epididymal epithelial cell structure in control mice was completed and the lumen was filled with sperm, **d** the epididymal epithelial cells in challenged mice were disorderly arranged, the number of sperm in the lumen was decreased. **e**–**j** TEM observation of testis in control (**e**,** f**) and challenged mice (**g**–**j**): **e**,** f** the secondary spermatocytes (**e**) and sperm cells (**f**) of control mice show regular aspects; **g–j** in challenged mice, TEM images show disolution of the nuclear membrane (**g**), swollen mitochondria and endoplasmic reticulum dilation (**h**), incomplete nuclear membrane and diverse mitochondrial morphology (**i**), membranous structure hyperplasia (**j**). **k**–**n** TEM observation of epididymis in control (**k**) and challenged (**l–n**) mice. **k** Dense microvilli, clear nuclear membrane and normal organelles in epididymis from control mice;** l**–**n** samples from infected mice showing shedded microvilli (**l**), decreased number of lysosomes, endoplasmic reticulum hyperplasia and swollen mitochondria with myelinated structure (**m**), sperm with two tails (**n**). Scale bars: 10 nm.* A* Acrosome,* EEC* epididymal epithelial cell,* ER* endoplasmic reticulum,* G* Golgi apparatus,* H* hyperplasia,* JC* junction complexes,* L* lysosome,* M* mitochondria,* Mi* microvilli,* NM* nuclear membrane,* S* sperm,* SC* spermatogenic cells

The testis and epididymis tissues of the challenged mice stained with HE staining were observed by light microscopy. The number and structure of spermatogenic cells in the testis of the mice in the control group were normal (Fig. 3a). Compared with the control group, the spermatogenic tubules of the testis of the challenged mice had thickened and became incomplete; the number of spermatogenic cells at all levels was significantly reduced, disorderedly arranged and seriously exfoliated (Fig. 3b). The epididymal epithelial cells were arranged neatly in the control group and an abundant amount of sperm were observed in the lumen (Fig. 3c). The epididymal epithelial structure in the challenged mice was damaged, and the epithelial cells were irregularly arranged and degenerated. There was a significantly reduced number of sperm in the lumen, or a large number of immature sperm, sperm remnants, deformed sperm; the most serious lesions included almost no sperm in the lumen. Vacuolation and degeneration of the cytoplasm and interstitial widening were also observed (Fig. 3d). The results indicated that *N. caninum* could induce damage to the testicular tissue and cells in mice.

### TEM observations of ultrastructural lesions

Ultrastructural observations indicated that the secondary spermatocytes in the control group were round with a smooth cell membrane, round nucleus and deeply stained (Fig. 3e). The sperm cells were round, with a smooth membrane, the nucleus was large and round, the double membranes were clear and the chromatin was compact (Fig. 3f). The structure of the mitochondria, endoplasmic reticulum, lysosome, Golgi apparatus and acrosome appeared normal in the cytoplasm. In the experimental (challenged) group, the membrane of the secondary spermatocytes was not clear, the perinuclear space was expanded, and the nuclear membrane was dissolved (Fig. 3g). The mitochondria showed slight swelling, cavitation, myelinated structure and endoplasmic reticulum dilation (Fig. 3h). There was a decreased number of sperm cells, the nuclear membrane was incomplete, there was a reduction in the number of organelles, diverse mitochondrial morphology and the number of mitochondrial crista was decreased (Fig. 3i). In the cytoplasm, membranous structure hyperplasia was observed, with possible development into myelin sheath and endoplasmic reticulum dilation (Fig. 3j); in addition, the cells exhibited a tendency towards degeneration and necrosis.

In the control group, the microvilli on the top of the chief cells of the epididymis were arranged in an orderly and dense manner. The nucleus was regular with heterochromatin, the nuclear membrane was clear and the nucleolus was prominent. The lysosome, rough endoplasmic reticulum and scattered mitochondria could be observed in the cytoplasm. There was a large number of mature sperm in the lumen and tight junction complexes between the cells (Fig. 3k). In the experimental group, the entire cell had become larger and electron density was increased. The microvilli on the top of the chief cell were sparsely arranged, and there was a large amount of shedding on the lumen. The tight junction complexes were slightly dissolved (Fig. 3l). There was a decreased number of lysosomes, a large amount of endoplasmic reticulum hyperplasia, swollen mitochondria with a myelinated structure and lesions of varying degrees (Fig. 3m). Sperm with two tails were occasionally observed in the lumen (Fig. 3n). The results showed that *N. caninum* could damage the structure of germ cells and induce cellular apoptosis at an early stage.

### Effect of *N. caninum* on the expression of p53 and caspase-3 in the testis

**Fig. 4:**
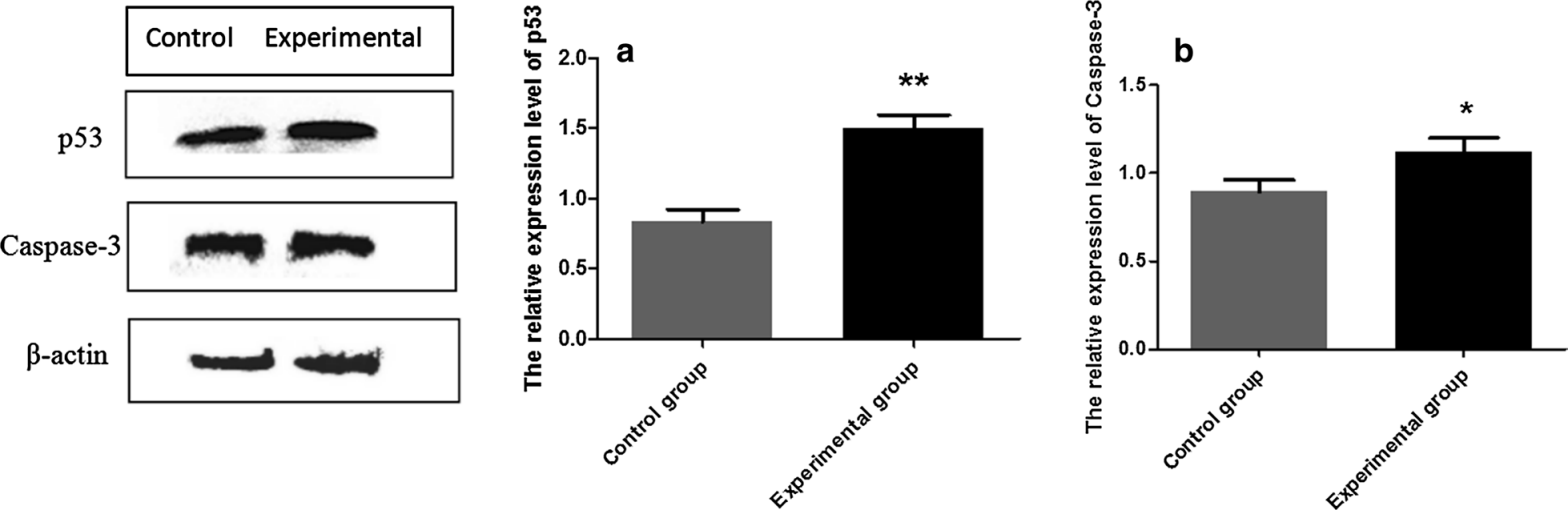
Western blot analysis of p53 and caspase-3 expression in testis of challenged and control male BALB/c mice. **a** Level of p53, **b** level of caspase-3. Data are presented as the mean ± SEM. Asterisks indicate that the difference between coefficients for the experimental group and control group are statistically significant at *P < 0.05 and **P < 0.01

The testis of the experimental mice was examined by western blot. The results showed that p53 expression was strongly upregulated in the testis compared with the control group (ANOVA: *F*_(1, 8)_ = 84.03, *P* = 0.001) (Fig. 4a) and that caspase-3 expression was significantly upregulated (ANOVA: *F*_(1, 8)_ = 14.74, *P* = 0.018) (Fig. 4b) in the testis of the experimental group.

### Effect of *N. caninum* on gene expression related to testicular spermatogenic function

**Fig. 5:**
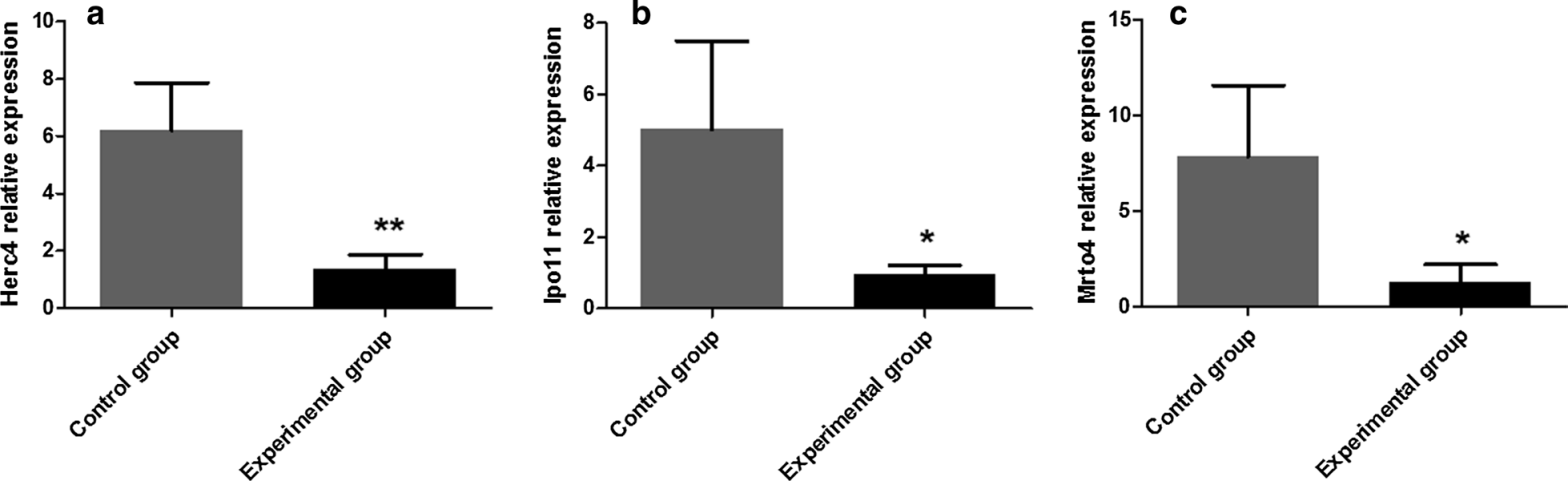
Relative expression levels of* Herc4* (**a**),* Ipo11* (**b**) and* Mrto4* (**c**) in challenged and control male BALB/c mice. The data are presented as the mean ± SEM. Asterisks indicate that the difference between the expression levels in the experimental group and control group are statistically significant at *P < 0.05 and **P < 0.01

The genes related to testicular spermatogenic function, namely* Herc4*,* Ipo11* and* Mrto4*, were detected by RT-PCR. The Cq values of the target gene and the reference gene were calculated using the appropriate software, and the relative level of the gene expression was calculated using the 2^-ΔΔCq^ formula. The results showed that compared with the control group,* Herc4* (ANOVA: *F*_(1, 8)_ = 22.49, *P* = 0.009) was extremely significantly reduced (Fig. 5a) and* Ipo11* (ANOVA: *F*_(1, 8)_ = 7.93, *P* = 0.048) and* Mrto4* (ANOVA: *F*_(1, 8)_ = 8.66, *P* = 0.042) were significantly decreased (Fig. 5b, c).

### Changes in sperm morphology and motility in challenged mice

**Fig. 6:**
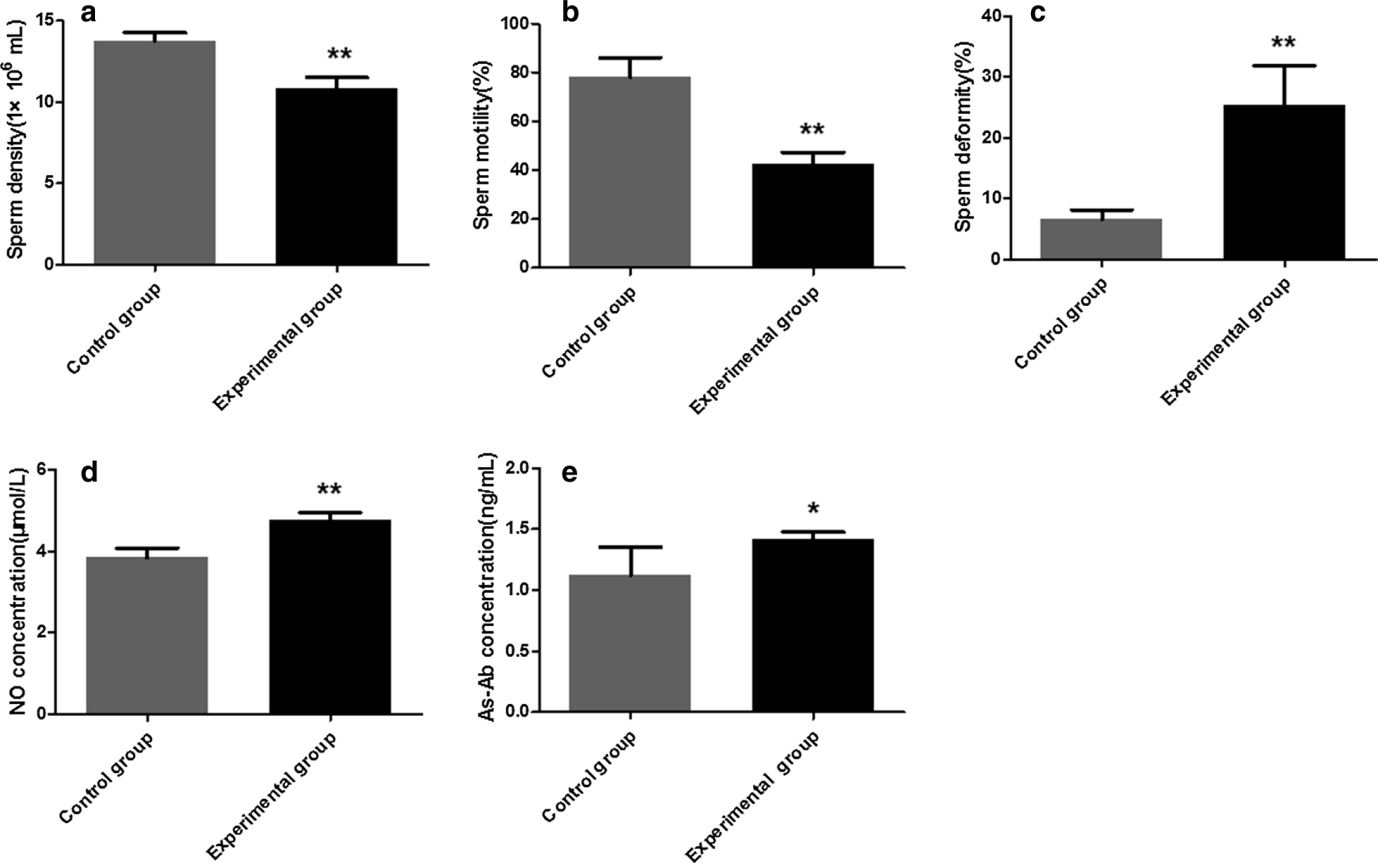
Density, motility, deformity rate, nitric acid (*NO*) and antisperm antibody (*AsAb*) of sperm in challenged and control male BALB/c mice. **a** Sperm density, **b** sperm motility, **c** sperm deformity, **d** NO concentration, **e** AsAb concentration. The data are presented as the mean ± SEM. Asterisks indicate that the difference between the values in the experimental group and control group are statistically significant at *P < 0.05 and **P < 0.01

A microscopic examination of the collected sperm showed that compared with the control group, sperm density (ANOVA: *F*_(1, 8)_ = 34.67, *P* = 0.004) and sperm motility (ANOVA: *F*_(1, 8)_ = 43.38, *P* = 0.003) were extremely significantly downregulated (Fig. 6a, b) and sperm deformities (ANOVA: *F*_(1, 8)_ = 24.40, *P* = 0.008) were strongly upregulated in the experimental group (Fig. 6c). The results of this study showed that the sperm from the mice in the experimental group was severely damaged.

### Effect of *N. caninum* on NO and AsAb levels in the testis homogenates

The level of NO and AsAb in the testicular homogenates was detected by ELISA. Compared with the control group, the level of NO (ANOVA: *F*_(1, 8)_ = 37.78, *P* < 0.0001) in the testis homogenate of the experimental group was extremely significantly increased (Fig. 6d) and the level of AsAb (ANOVA: *F*_(1, 8)_ = 6.30, *P* = 0.04) was significantly increased (Fig. 6e); both results indicate abnormal spermiogenesis function.

### Effect of *N. caninum* on serum hormone levels

**Fig. 7:**
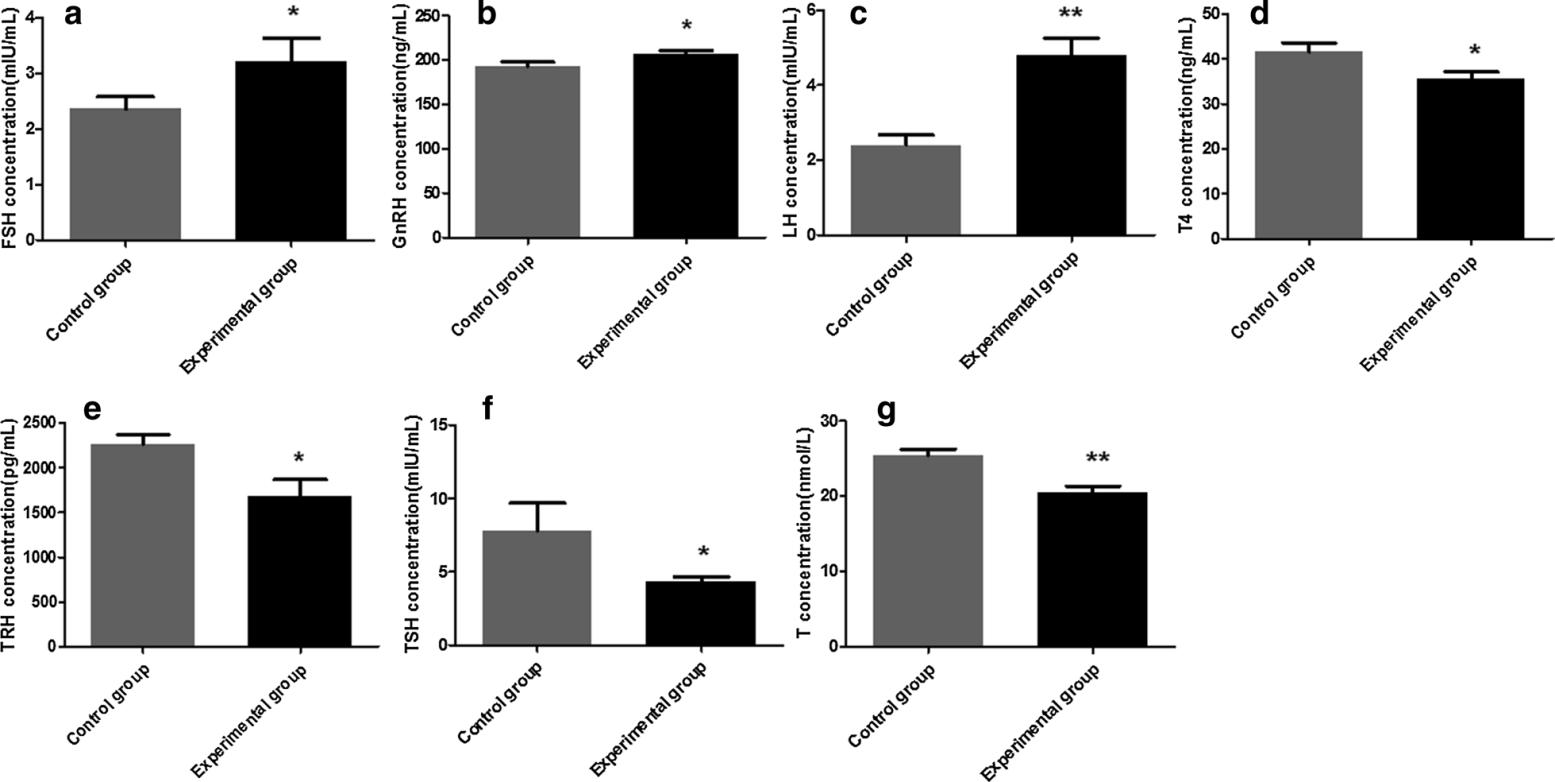
Hormones levels in challenged and control male BALB/c mice. **a** follicle-stimulating hormone (*FSH*), **b** gonadotropin-releasing hormone (*GnRH*), **c** luteinizing (*LH*), **d** thyroxine (*T4*), **e** thyrotropin-releasing hormone (*TRH*), **f** thyroid-stimulating hormone (*TSH*) and **g** testosterone (*T*). The data are presented as the mean ± SEM. Asterisks indicate that the differences between the concentration of the hormones in the experimental group and control group are statistically significant at *P < 0.05 and **P < 0.01

Compared with the control group, the levels of GnRH, FSH and LH in the experimental group were upregulated (Fig. 7a–c) and the levels of TRH, T4, TSH and T were downregulated (Fig. 7d–g), as determined by ELISA. The levels of LH (ANOVA: *F*_(1, 8)_ = 69.17, *P* < 0.0001) and T (ANOVA: *F*_(1, 8)_ = 61.83, *P* < 0.0001) in the experimental group were extremely significantly different from those in the control group, and the levels of GnRH (ANOVA: *F*_(1, 8)_ = 10.52, *P* = 0.032), FSH (ANOVA: *F*_(1, 8)_ = 9.86, *P* = 0.02), TRH (ANOVA: *F*_(1, 8)_ = 12.62, *P* = 0.012), T4 (ANOVA: *F*_(1, 8)_ = 14.80, *P* = 0.018) and TSH (ANOVA: *F*_(1, 8)_ = 9.76, *P* = 0.026) in the experimental group were significantly different. These results indicate that *N. caninum* could induce parasecretion of hormones in the male mice, which in turn affected hypothalamic pituitary testicular axis function.

### Effect of challenged mice on reproductive capacity

**Fig. 8:**
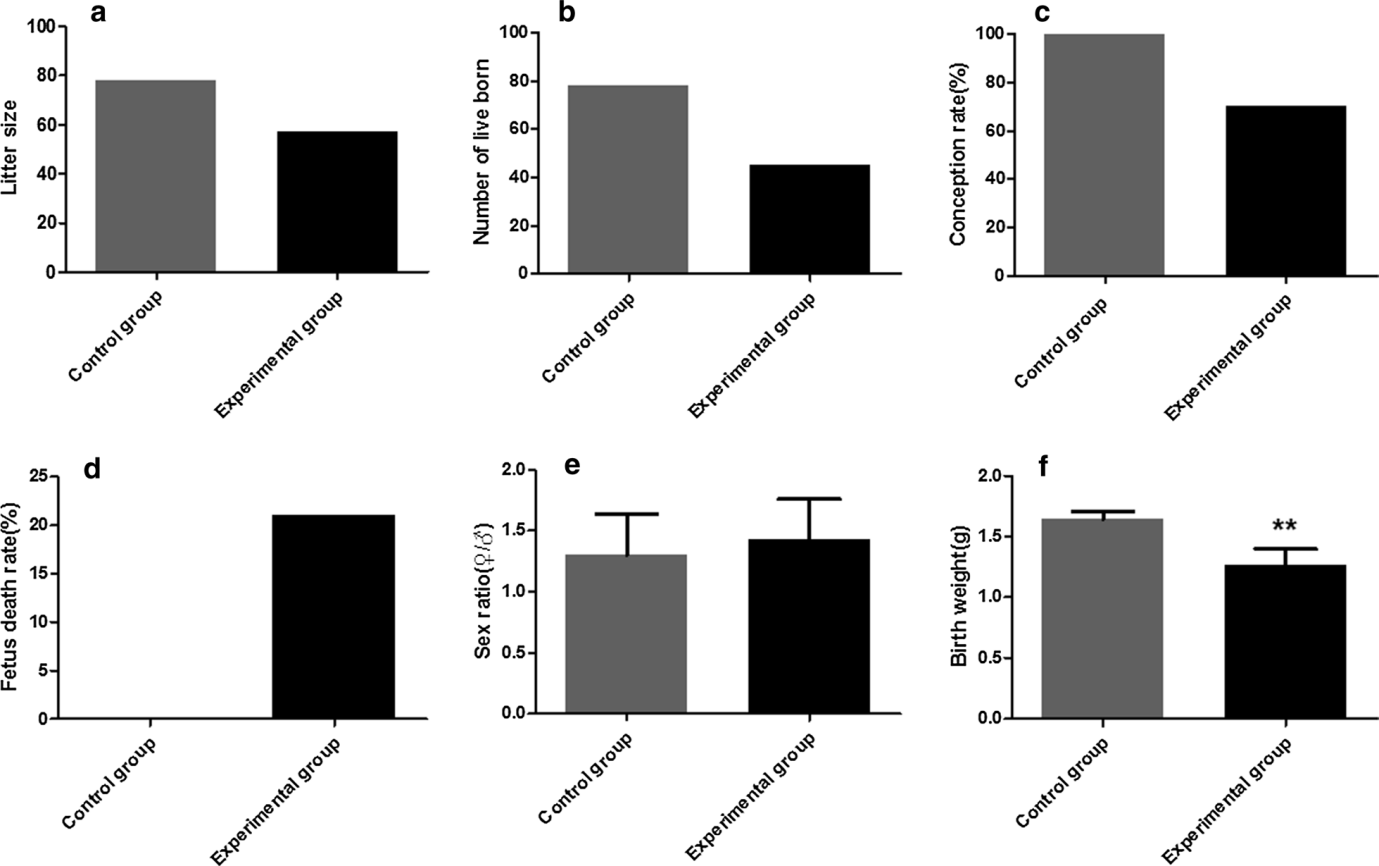
Reproductive capacity indexes in challenged and control male BALB/c mice. **a** Litter size, **b** number of live birth, **c** conception rate, **d** fetus death rate, **e** sex ratio, **f** birth weight. The data are presented as the mean ± SEM. Double asterisk indicates that the difference between the index of the experimental group and control group is statistically significant at **P < 0.01

The male mice in the model and in the control groups were caged with female mice, respectively. The results showed that the litter size in the experimental group was 57 (Fig. 8a), the number of live born was 45 (Fig. 8b) and the conception rate was 70% (Fig. 8c); all values were significantly lower than those of the control group. The fetus death rate in the experimental group and control group was 21.05 and 0%, respectively (Fig. 8d). There was no statistically significant difference in the female/male sex ratio of F1 offspring (ANOVA: *F*_(1, 123)_ = 0.61, *P* = 0.45) (Fig. 8e). Compared with the control group, the birth weight was extremely significantly reduced (ANOVA: *F*_(1, 123)_ = 60.20, *P* < 0.0001) (Fig. 8f).

## Discussion

*Neospora caninum* is an obligate intracellular parasite that primarily causes abortion and stillbirth, as well as neonatal dyskinesia and nervous system diseases [13]. Many aspects of *N. caninum* infection remain unclear, such as the pathogenic mechanism, relationship between the distribution of the parasite in the host and clinical symptoms. Zhou et al. investigated the mechanism of *Toxoplasma gondii* on male reproductive injury and confirmed that it reduced the vitality and survival rate of sperm and also damaged the reproductive system [14]. *Neospora caninum* and *Toxoplasma gondii* are similar in many respects. To date, little research has focused on the damage to the male reproductive system caused by *N. caninum*. Therefore, this study on the effect of *N. caninum* on damage to male reproductive function is of great significance for exploring diseases of the reproductive system and methods of prevention.

Apoptosis is a process of active cell death under the control of the apoptosis-related genes [15]. Under normal circumstances, apoptosis can regulate the stability of the internal environment; however, following parasitic infection, spermatogenic cells in the body exhibit evidence of excessive apoptosis. It has been found that the p53 gene can induce cell senescence and apoptosis and control the quality of sperm [16]. Caspase-3 can be activated by a variety of stimulating factors, cleave proteins, hinder DNA replication and cell repair, destroy the entire nuclear structure and induce apoptosis [17]. In our study, the western blot analysis showed that the levels of p53 and caspase-3 protein expression in the testis and epididymis of challenged mice were significantly upregulated. These results indicate that *N. caninum* infection can promote spermatogenic cell apoptosis and affect the reproductive function of male mice.

The* Herc4*,* Mrto4* and* Ipo11* genes are closely related to the production and functional expression of sperm.* Herc4* exhibits ubiquitin ligase activity and is involved in the development and maturation of sperm in male mammals [18]. * Mrto4* can adjust the polyadenylation and deadenylation of some special mRNAs during spermatogenesis [19].* Ipo11* is an important gene that influences the expression of tumor suppressor gene *TSLC1/IGSF4*. *TSLC1/IGSF4* has been shown to promote the differentiation and maturation of spermatogenic cells and spermatozoa [20]. Changes (e.g. deletion, translocation, upregulation or downregulation) of these genes could affect the normal spermatogenesis process. The results of this study demonstrate that* Herc4* expression in the challenged mice was extremely significantly reduced, whereas the expressions of* Ipo11* and* Mrto4* were significantly reduced. This finding suggests that *N. caninum* has substantial influence on the physiological function of germ cells and could lead to the occurrence of sterility.

The success rate of fertilization is dependent on whether the sperm function and structure are complete, which is directly reflected by sperm motility. Several factors have been shown to cause sperm dysfunction and structural abnormalities [21]. The density and motility of sperm and the number of deformities are physiological constants that have been found to directly reflect male reproductive function [14]. The results of the present study indicate that sperm density and sperm motility were extremely significantly reduced, whereas sperm deformities were strongly increased in the experimental group, suggesting suggested that *N. caninum* affected the quality of semen and spermatogenic function in the challenged male mice. NO is an active molecule that regulates biological function and is widely expressed in the mammalian reproductive system. NO has been reported to affect spermatogenesis and regulate androgen secretion [22]. Moreover, previous studies have shown that NO has a biphasic effect on steroid secretion; lower doses of NO stimulate testosterone secretion, while higher doses inhibit testosterone secretion, thereby inhibiting sperm production [23]. Our results show that the level of NO in the testis homogenate in the experimental group was extremely significantly increased, suggesting that *N. caninum* can cause testicular tissue injury. AsAb represents an autoantibody in male animals, which can lead to male infertility by reducing sperm survival rate and motility [24]. Our results show that the level of AsAb in the testis homogenate in the experimental group was significantly increased, indicating that *N. caninum* can reduce the survival rate, motility and fertilization rate of sperm by increasing the concentration of AsAb, thereby affecting the reproduction ability of male animals.

The normal reproductive function of males is regulated by sex hormones, and the secretion of sex hormones is regulated by the hypothalamic–pituitary–gonad (HPG) axis. The HPG includes GnRH, LH, FSH and sex steroids [25]. These hormones can both affect the normal development and function of the gonads, as well as a feedback effect, which could regulate the reproductive circuit of the brain to achieve an overall regulation of the HPG axis [26]. A series of stresses and metabolic responses during the growth and development of the body are regulated by three endocrine systems: hypothalamic–pituitary–adrenal (HPA) axis, HPG axis, and hypothalamic–pituitary–thyroid (HPT) axis. An appropriate amount of thyroid hormones is beneficial to the stability of the HPG axis; however, abnormal levels of thyroid hormones could affect the HPG axis through different means, resulting in disorders of sex hormone secretion and gonadal function [27]. The results of this study show that the levels of LH, FSH and GnRH were significantly increased, whereas the levels of T, TRH, T4 and TSH were significantly decreased in the experimental group. *Neospora caninum* induced disordered hormone secretion and abnormal hypothalamic–pituitary–testicular axis function in male mice, which further affected reproductive function. However, the specific impact mechanism is still unclear.

 Masuda et al. intraperitoneally inoculated CB-17 SCID and BALB/c male mice with *N. caninum* and found that immunodeficient pregnant mice were more likely to transmit tachyzoites to fetal mice by venereal transmission while immunocompetent female mice did not transmit tachzoites to fetal mice [28]. However, based on their results, of Masuda et al. assumed that *N. caninum* still had an impact on the reproductive capacity and offspring of male mice. Therefore, in our study, male mice infected with *N. caninum* were caged with normal female mice. Our results show that the litter size, number of live births and conception rate in the experimental group were significantly reduced compared with the control group. The birth weight in the experimental group was significantly reduced, indicating that an *N. caninum* infection could lead to a reduction in the reproductive capacity of male mice.

## Conclusions

Using a male BALB/c mouse model of *N. caninum* infection, we investigated the organ coefficients of the testis and epididymis, pathology, expression of spermatogenic cell apoptosis-related proteins and spermatogenesis-related genes, level of sperm quality, presence of AsAb, NO and hormone levels, as well as reproduction. Our findings demonstrate that *N. caninum* could induce varying degrees of damage to the testis, epididymis, sperm and other cells of the male mice, cause hormone imbalance, inhibit sperm production and affect the reproductive ability. Thus, these findings provide a basis for the further study of the pathogenic mechanisms of *N. caninum*.

## Data Availability

All data analyzed or generated during this study are included in this published article.

## Abbreviations

AsAb: Antisperm antibody
ELISA: Enzyme-linked immunosorbent assay
FSH: Follicle-stimulating hormone
GnRH: Gonadotropin-releasing hormone
HE: Hematoxylin and eosin
HPA: Hypothalamic–pituitary–adrenal
HPG: Hypothalamic–pituitary–gonadal
HRP: Horseradish peroxidase
HPT: Hypothalamic–pituitary–thyroid
LH: Luteinizing hormone
NO: Nitric oxide
RIPA: Radio-immunoprecipitation assay
SDS-PAGE: Sodium dodecyl sulfate–polyacrylamide gel electrophoresis
T4: Thyroxine
T: Testosterone
TRH: Thyrotropin-releasing hormone
TSH: Thyroid-stimulating hormone
